# Prevalence of torque teno virus in healthy individuals and those infected with hepatitis C virus living in Yazd, Iran

**DOI:** 10.22088/cjim.11.2.199

**Published:** 2020

**Authors:** Mansour Moghimi, Mohammad Shayestehpour, Masoud Doosti, Abbas Ahmadi Vasmehjani, Seyed Mahmood Seyed khorrami, Akram Sadat Ahmadi, Mohsen Akhondi-Meybodi

**Affiliations:** 1 Department of Pathology, School of Medicine, Shahid Sadoughi University of Medical Sciences, Yazd, Iran; 2 Autoimmune Diseases Research Center, Kashan University of Medical Sciences, Kashan, Iran; 3 Department of Microbiology and Immunology, Faculty of Medicine, Kashan University of Medical Sciences, Kashan, Iran; 4 Infectious and Tropical Diseases Research Center, Shahid Sadoughi University of Medical Sciences, Yazd, Iran; 5 Department of Medical Virology, Faculty of Medical Sciences, Tarbiat Modares University (TMU), Tehran, Iran; 6 Virology Department, School of Public Health, Tehran University of Medical Sciences, Tehran, Iran; 7 Gastroentrology Department, Shahid Sadoughi Hospital, Faculty of Medicine,, Shahid Sadoughi University of Medical Sciences,Yazd, Iran

**Keywords:** Genotype, Hepatitis C, Prevalence, Torque teno virus

## Abstract

**Background:**

Torque teno virus (TTV) is a non-enveloped DNA virus that its role as a helper or causative agent in hepatitis is still unclear. TTV prevalence varies in different regions of the world. This study aimed to determine the prevalence of TTV in healthy individuals and those infected with hepatitis C virus (HCV) living in Yazd city, Iran.

**Methods:**

In this case-control study, 50 healthy subjects and 68 HCV-positive individuals who referred to Yazd hospitals participated in this study. TTV DNA in serum samples were detected by nested polymerase chain reaction (PCR) using specific primers of 5΄-UTR and N22 regions. The genotypes of HCV and TTV were determined by sequencing method.

**Results:**

TTV-DNA was detected in 2 out of 50 (4℅) healthy individuals and in 4 out of 68 (5.8℅) HCV-positive persons. There was not a significant correlation between the prevalence of TTV and HCV infection. The most common TTV genotypes among HCV-positive individuals were 3, 17 and 13, respectively. There was not a significant association obtained between HCV genotypes and TTV genotypes.

**Conclusion:**

The prevalence of TTV in Yazd province was low compared with the other areas of Iran. The prevalence of TTV in HCV infected people was not significantly higher than its rate in uninfected individuals.

Torque teno virus or transfusion transmitted virus (TTV) is a non-enveloped virus with single stranded circular DNA genome that is currently classified in the Alphatorquevirus genus and the family of Anelloviridae (1). TTV was first isolated in 1997 from serum of a Japanese patient with non A-G hepatitis who had history of blood transfusion (2). Unlike other DNA viruses, TTV has an extremely wide genomic diversity, so that five main clades and more than 35 genotypes have already been detected. Unexpected high mutation rate in TTV DNA could be due to recombination, intragenomic rearrangement or the presence of hypervariable regions in ORF1 (3). In the past, blood transfusion was considered as the only transmission route of TTV, but today it is suspected to be transmitted through intravenous drugs, sexual, maternal and fecal-oral routes (4). The prevalence of TTV ranged from 1 to 34℅ among healthy people in developed countries and from 40 to 70℅ in the developing countries (5) based on molecular tests. The seroprevalence of TTV among adults and children 2–4 years is reported 42℅ and 43℅, respectively. TTV13 species-specific IgG was predominated in adults, whereas TTV13 IgG was predominated in children (6). Some researchers have observed a high seroprevalence of TTV up to 98.6% in hepatitis patients with unknown etiology as well as in blood donors (7). 

Previous studies have shown that torque teno virus is hepatotropic and replicates in liver. The titer of TTV genome has been reported 10-100-fold higher in liver tissue than in serum. TTV DNA is detected in 47℅ of patients suffering from fulminant hepatic failure and in 46% of patients with chronic hepatitis of unknown etiology (8). Nevertheless, the role of TTV in acute and chronic liver diseases remains unclear and controversial (9). 

Some studies have evaluated the association between TTV, hepatitis B virus (HBV) and hepatitis C virus (HCV) infections (4, 10, 11). Researchers have found the high *TTV* viral load as a significant *risk factor* for hepatocellular carcinoma (HCC) in people with HCV. HCV/TTV co-infection *can result in* a higher histological grade score compared to *HCV infection without TTV (*12*).*

*Variations in nucleotide sequence of TTV genotypes have led to create different genotypes. In the majority of studies, *TTV genotypes have been determined using N22 region of viral genome. Recent reports have used untranslated region (UTR) of viral genome for genotyping (13). The UTR region is conserved and contains sequences with more than 90% identity among all genotypes. UTR primers can detect more TTV genotypes in comparison with N22 primers. Phylogenetic analysis performed on the UTR region is more accurate (4). 

In recent years, researchers have focused on the prevalence and genotyping of TTV in healthy individuals and HCV infected peoples. Data published on circulating TTV genotypes in different areas of Iran are low; therefore, the present study aimed to estimate the prevalence of TTV in HCV infected and uninfected people and determine TTV genotypes in HCV-positive peoples living in Yazd, Iran. 

## Methods

**Study population: **In this case-control study, 68 people with HCV infection and 50 healthy individuals who referred to hospitals in Yazd city participated. Individuals with a positive-PCR result for HCV were included in the study as case while people with negative-HCV-PCR test were considered as control (sex and age-matched). Subjects with liver disorder, autoimmune diseases, cancer, HBs-Ag, HIV-Ab and HCV-Ab were excluded from the control group. Three milliliters of blood sample were collected from the subjects. Serum was separated and cryopreserved at -70°C until analysis time. Demographic data were collected by a questionnaire. This study was approved by the Ethics Committee of Shahid Sadoughi University of Medical Sciences-Yazd (Ethical code: IR.SSU. SPH.REC.1391.181964. All subjects completed the informed consent forms.

**TTV detection** **through PCR and genotype determination via sequencing: **Viral DNA was extracted from sera using the High Pure Viral Nucleic Acid kit (Roche Diagnostics, Mannheim, Germany) according to manufacturer's protocol. Then, nested polymerase chain reaction (Nested PCR) was performed using primers, which replicated 5’-UTR or N22 region of TTV genome (table 1). 

**Table 1:** Primer sequences for detection of torque teno virus

**Primer **	**Region**	**Primer Sequence**
NG 054	5’-UTR	5΄- TTT GCT ACG TCA CTA ACC AC -3΄
NG1471	5’-UTR	5΄-GCC AGT CCC GAG CCC GAA TTG CC-3΄
NG1321	5’-UTR	5΄-AGC CCG AAT TGC CCC TTG AC -3΄
NG059	N22	5΄-ACA GAC AGA GGA GAA GGC AAC ATG-3΄
NG061	N22	5΄-GGC AAC ATG YTR TGG ATA GAC TGG-3΄
NG063	N22	5΄-CTG GCA TTT TAC CAT TTC CAA AGT T-3΄

The reaction mixture of first round of nested PCR test for TTV-N22 region contained 10 μL of template DNA, 0.5 μL primer NG059 (10pM stock), 0.5 μL primer NG061(10pM stock), 12.5 μL of the master mix (Sinaclon, Tehran, Iran) and 1.5 μL of DEPC water. The second round of reaction was performed using 10 μL of the first product, 0.5 μL of primer NG059 (10pM stock), 0.5 μL primer NG063 (10pM stock) 12.5 μL of the master mix (Cinnagen, Tehran, Iran) and 1.5 μL of DEPC water. PCR amplification was performed with an initial denaturation at 94 °C for 7 min followed by 40 (for the first round) or 35 (for the second round) cycles of 30 sec at 94 °C, 45 sec at 60 °C, and 1 min at 72 °C, with a final extension at 72 °C for 5 min. The PCR products were run on 2% agarose gel by electrophoresis, stained with DNA safe stain (Cinnagen, Tehran, Iran), and visualized using a gel documentation system. The amplification fragment of the second step of PCR was 271 bp. 

The nested PCR reaction for TTV-5’UTR region was carried out with same volumes and reaction contents noted for the N22. NG054 and NG1471 primers were used in the first step of PCR, and the second round was performed using NG054 and NG1321 primers. The amplification was carried using temperature conditions as follows: initial denaturation at 94 °C for 5 min, 35 (for the first round) or 25 (for the second round) cycles of 94°C at 45 sec, 60°C at 45sec, 72°C at 45 sec with and final extension at 72°C for 5 min. The amplification fragment of the second step of PCR was 220bp. Nested PCR products of TTV-N22 and TTV-5’UTR regions purified and sequenced using a DNA sequencing system and BigDye® Terminator v3.1 Cycle Sequencing Kit (Applied Biosystems Foster City, CA) according to the manufacturer’s protocols. TTV genotypes were determined by the alignment of PCR product sequences with the sequences of TTV genotypes, which were obtained from GenBank. Phylogenetic analysis was performed based on the 5' untranslated region of TTV genome reference sequences by MEGA7 software and neighbor-joining (NJ) method. Phylogenetic tree was constructed with 500 bootstrap repeats. Statistical analysis was carried out using Student’s t-test, and chi-square test by SPSS software Version 22. A pvalue less than 0.05 was considered significant.

## Results

Table 2 shows the subjects' characteristics and the result of the laboratory tests. Serum samples of 118 participants (74 men and 44 women) including 68 individuals with HCV infection and 50 healthy subjects were screened by PCR test for finding the TTV genome using specific primers for 5’-UTR and N22 regions. 

**Figure 1.**
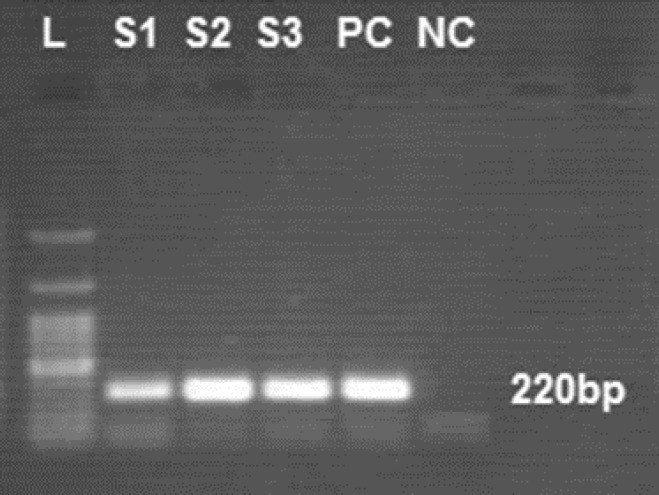
Detection of torque teno virus (TTV) by polymerase chain reaction of the 5' untranslated region of genome. Lane 1 (L), 100 bp DNA ladder; lanes 2 to 4 (S1, S2, S3), positive samples of TTV; lanes 5 (PC) and 6 (NC), positive and negative controls, respectively

TTV-DNA was detected in 4 out of 68 (5.8%) HCV-positive individuals and in 2 out of 50 (4%) healthy individuals (fig 1).

**Table 2:** Patient’s characteristics and results of laboratory tests

**Characteristic**		**Number (%)**		**Pvalue**
		**TTV** ^+^	**TTV** ^-^	
Age group (years)	≤30	4 (7)	53 (93)	0.846
	30-40	2 (4.2)	46 (95.8)	
	40-50	0	9 (100)	
	≥50	0	4 (100)	
Sex	Male	1 (1.4)	73 (98.6)	0.027
	Female	5 (11.4)	39 (88.6)	
HCV infection	Positive	5 (7.4)	63 (92.6)	0.240
	Negative	1 (98)	49 (2)	
Aminotransferases	ALT	57±25.47	33.5±10.66	0.043
	AST	44±13.79	44.04±21.59	0.996

**Figure 2:**
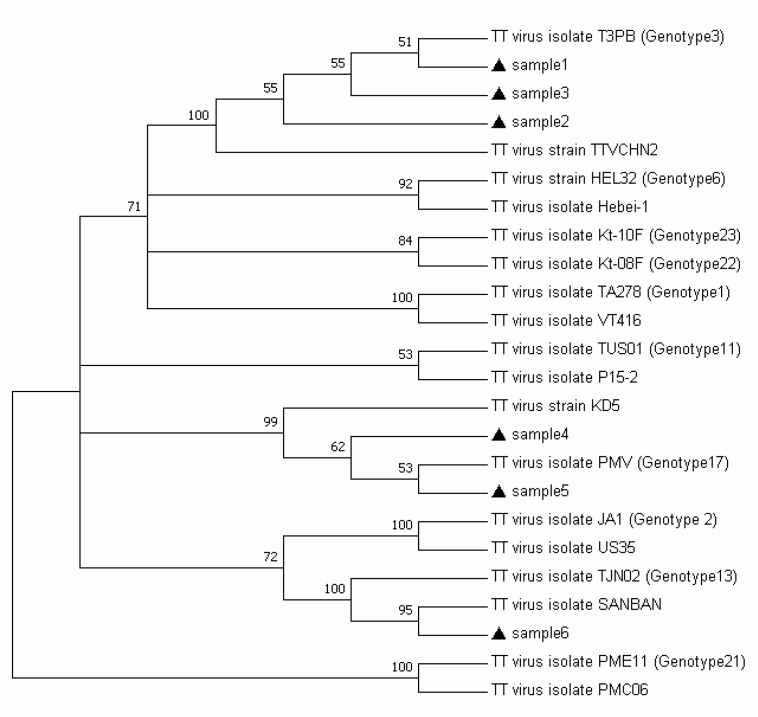
Phylogenetic tree of TTV constructed by the neighbor-joining method, Based on 5’-UTR sequence. Black triangles denote samples under study

These data did not show a significant correlation in the prevalence of TTV between case and control group (p=0.240). ALT in TTV positive subjects was significantly higher than those negative for TTV (p=0.043), but AST did not show a significant difference between the two groups. Of the 68 individuals with HCV, 63.2% had genotype 1a and 36.8% were identified for genotype 3a. The most frequent HCV genotype in TTV-positive subjects was 1a. There was not a correlation observed between HCV genotypes and HCV/TTV coinfection (p=0.645). Phylogenetic analysis was performed (Fig 2) and only three genotypes of TTV were detected in HCV-positive persons (table 3). The most common genotypes were 3, 17 and 13, respectively. The different TTV genotypes were not associated with HCV genotypes (p=0.858). 

**Table 3:** Genotype distribution of TTV in people infected with HCV

**Result**		**TTV result (%)**				**P value**
		**Genotype 3**	**Genotype17**	**Genotype 13**	**Negative**	
HCV genotype	1a	2 (4.7)	2 (4.7)	1 (2.3)	39 (88.3)	0.858
	3a	1 (4)	0	0	24 (96)	
Total	-	3 (4.3)	2 (2.9)	1 (1.5)	63 (91.3)	

## Discussion

TTV was isolated for the first time from a cryptogenic hepatitis patient, therefore, assumed that its pathogenicity could be associated with hepatitis (14). Some studies have significantly reported higher rates of TTV infection in HCV-positive persons compared with healthy individuals (4, 15, 16), but the role of TTV as a helper or causative agent in hepatitis is still unclear. 

In the present study performed in Yazd, the prevalence of TTV-DNA in healthy subjects and HCV infected individuals was 4% and 5.5%, respectively. The rate of TTV among the healthy population in Yazd is lower than the other reports in IRAN. The prevalence of TTV has been estimated to be 66.9% in Tehran, 23.7% in Ahvaz, 92.5% in Shiraz (4), and 41% in Isfahan (17). The rate of TTV in the southwest of Iran was reported to be low (2.9%) (18) similar to the result of the current study. 

In this study, the results did not show a significant correlation between the prevalence of TTV and HCV infection (P=0.240). The association between TTV and HCV is still controversial. Some researchers have found a correlation between these two viruses, while the others could not show any relationship. The prevalence of TTV in the USA and European countries has reported about 1%. In Pakistan, TTV prevalence was reported 3.6% in HCV positive persons and 3.2% in healthy individuals (19). That is in range of the current study. In contrast with the result of the present study, some studies conducted in Iran have found a correlation between the prevalence of TTV and HCV infection. 

In a study performed in Jahrom, by Mousavinasab et al., 18% of the healthy subjects and 66.5% of HCV-positive individuals were positive for TTV (16). In the study of Kenarkoohi et al., the prevalence of TTV in HCV-positive persons living in Shiraz was more than 90% (4). TTV was detected in 65% of healthy people and 67.5% of HCV-infected individuals in east Azerbaijan province (15); therefore, similar to our finding, the prevalence of TTV in HCV-positive people was not significantly more than the HCV-negative subjects. 

Several factors, including geographical distribution of population, sample size and the diagnostic methods can make the variability in the results of TTV prevalence. The primers for detecting of TTV in our study and studies noted above were designed for the same region of viral genome (5'UTR and N22); therefore, it can be concluded that the prevalence of TTV in Yazd province is significantly lower than in Jahrom, Shiraz and east Azerbaijan among the HCV-positive or negative people. There was not any report found in the literature review on the TTV prevalence in Yazd. 

TTV is a DNA virus, but unusual genetic variability occurs in its genome. The presence of animal carriers of the virus and the genetic recombination of viruses among human or human with animal may contribute in making diversity in TTV genome. Five main clades and more than 35 genotypes have already been determined for TTV. In the present study, genotypes 3, 17 and 13 were found in HCV-positive group, and we did not determine TTV genotypes in the control group. Kenarkoohi et al. detected genotypes 1, 3, 17, and 22 in people infected with HCV and genotype 11 was predominant (4). 

In a study conducted in Qatar, the most frequent genotypes isolated from HCV-positive individuals were 2, 3, 5, and 1, respectively (3). Previous studies showed that the dominant TTV genotypes worldwide are 1 and 3, while in Iran is 2 (4). The result of the present study did not show a significant association between HCV and TTV genotypes. This finding may be due to the low prevalence of TTV among our study population, but the previous studies also did not find a correlation between genotypes of these two viruses (3). 

Finally, the prevalence of TTV in Yazd province is low in comparison with other areas of Iran. The prevalence of TTV in HCV-positive individuals is not significantly higher than its rate in healthy people. The most common TTV genotypes were 3, 17 and 13, respectively. Further studies with more sample size are required to confirm these findings in Yazd province of Iran.
